# Relationship between EMG and fNIRS during Dynamic Movements

**DOI:** 10.3390/s23115004

**Published:** 2023-05-23

**Authors:** Natalia Daniel, Kamil Sybilski, Wojciech Kaczmarek, Dariusz Siemiaszko, Jerzy Małachowski

**Affiliations:** 1 Faculty of Mechatronics, Armament and Aviation, Institute of Rocket Technology and Mechatronics, Military University of Technology, 2 gen. S. Kaliskiego Street, 00-908 Warsaw, Poland; 2 Faculty of Mechanical Engineering, Institute of Mechanics & Computational Engineering, Military University of Technology, 2 gen. S. Kaliskiego Street, 00-908 Warsaw, Poland; 3 Department of Functional Materials and Hydrogen Technology, Military University of Technology, 2 gen. S. Kaliskiego Street, 00-908 Warsaw, Poland

**Keywords:** EMG, oxygen consumption, fNIRS, signal changes, correlation

## Abstract

In the scientific literature focused on surface electromyography (sEMG) and functional near-infrared spectroscopy (fNIRS), which have been described together and separately many times, presenting different possible applications, researchers have explored a diverse range of topics related to these advanced physiological measurement techniques. However, the analysis of the two signals and their interrelationships continues to be a focus of study in both static and dynamic movements. The main purpose of this study was to determine the relationship between signals during dynamic movements. To carry out the analysis described, the authors of this research paper chose two sports exercise protocols: the Astrand–Rhyming Step Test and the Astrand Treadmill Test. In this study, oxygen consumption and muscle activity were recorded from the gastrocnemius muscle of the left leg of five female participants. This study found positive correlations between EMG and fNIRS signals in all participants: 0.343–0.788 (median-Pearson) and 0.192–0.832 (median-Spearman). On the treadmill, the signal correlations between the participants with the most active and least active lifestyle achieved the following medians: 0.788 (Pearson)/0.832 (Spearman) and 0.470 (Pearson)/0.406 (Spearman), respectively. The shapes of the changes in the EMG and fNIRS signals during exercise suggest a mutual relationship during dynamic movements. Furthermore, during the treadmill test, a higher correlation was observed between the EMG and NIRS signals in participants with a more active lifestyle. Due to the sample size, the results should be interpreted with caution.

## 1. Introduction

Electromyography (EMG) records the electrical activity of action potentials that initiate muscle contraction and force production [1]. In the last 20 years, the number of surface EMG devices (sEMG) has increased significantly [2,3,4]. sEMG devices are commonly used in research in electrodiagnostic medicine, rehabilitation, sports science, kinesiology, ergonomics, and clinical neurophysiology [5,6]. Most researchers have started to precisely monitor changes in human muscle activity during various tests and have also helped to experimentally identify its movements and activity [7,8].

In addition to EMG, optical measurement methods are available and are well known for analyzing muscle work. One such method is near-infrared spectroscopy (NIRS), which allows continuous monitoring of a muscle during motor activity or rehabilitative exercises [9,10]. NIRS takes advantage of the fact that light in the spectral range between 600.0 and 1000.0 nm (the ‘therapeutic window’) can penetrate biological tissues up to a few centimeters completely without invasiveness [11]. Infrared light is absorbed by oxygenated hemoglobin (O2Hb) and deoxygenated hemoglobin (HHb), and the absorbance adjusted using the modified Beer–Lambert law is used to calculate the concentration of chromophores (i.e., O2Hb and HHb) [12]. fNIRS technology allows changes in muscle metabolism and muscle oxygenation to be observed during and after exercise or training interventions, both in the laboratory and during competition [13]. Numerous studies have implemented fNIRS devices in specific protocols focused on running [14], cycling [15], or even swimming [16]. The method of signal interpretation depends on the needs of the individual study, the availability of real-time signals, and the type of measurement: rest, exercise, or recovery. During rhythmic exercise, the action of the muscle pump influences the blood vessels to cause volumetric changes, which can be visualized as cyclic changes in the O2Hb and HHb signals that correspond, for example, to the step action [16].

The sEMG and fNIRS methods are used separately or together. In articles on sports activity and neurophysiology, the focus has been on various sports disciplines as subjects of research [17,18]. Different muscles are examined according to the type of disease (injury) or the sport activity performed. Furthermore, the methods used to interpret specific signals depend on the needs of the study and can be modified for data analysis or processing [19].

On the basis of the state-of-the-art survey conducted, it can be easily stated that there are a limited number of studies that are concerned with the problem of motion exercises (dynamic movement). Smith et al. [20] used fNIRS Oxymon MKIII (Artinis) and EMG Bagnoli (DelSys Inc., Boston, MA, USA) in their studies. They analyzed the endurance of 13 athletes from a team sport during cycling sprints. The influence of cerebral oxygenation (prefrontal cortex) and muscle (vastus lateralis) on the ability to perform repeated tests was examined. Di Giminiani et al. [21] compared regional muscle oxyhemoglobin saturation and surface EMG data measured under resting and dynamic conditions (treadmill run and strength exercises). They implemented a recently developed integrated quadriceps muscle oximetry/EMG system. In addition, many of the available articles focused on the implementation of fNIRS and EMG technologies in motion but were not related to the interrelationship of signals during specific sports in dynamic movements. Moreover, most of them do not include a description of the methods of signal analysis.

Finding a relationship between fNIRS and EMG signals was the main concern of the state-of-the-art survey conducted, and studies that previously compared this [22] first showed a linear relationship between VO2 and EMG under isometric conditions. However, the results of the experimental tests revealed that an accurate evaluation of both signals can be caused by technical problems related to the unambiguous mounting of sensors under dynamic conditions (possibilities of sensor movements during motion, anatomical differences, sweating, etc.). Furthermore, the time courses and relationships between oxygenation, blood volume, and vastus lateralis activity in children were investigated in submaximal isometric exercise [23]. The results showed a relationship between the changes in EMG and fNIRS and their ability to analyze and evaluate the local electrophysiological and metabolic characteristics of muscle adaptation in healthy children. The authors advised carefulness when interpreting the data and suggested the development of research in this area. Another line of research looked at the comparison of fNIRS and sEMG in terms of fatigue in isometric contraction [24], which described the analysis and correlation of parameters between activity and local tissue oxygenation using protocols to determine voluntary fatigue (compared to fixed exercise time). Finally, one of the most recent studies that analyzed muscles at the moment of hand flexion [25] showed calculated correlation coefficients, which only relate to acceleration (ACC) and EMG (static 0.18, dynamic 0.22). In terms of comparing fNIRS and EMG signals, the authors of the article presented results that showed that as the EMG amplitude increased, there was a parallel increase in the concentration of deoxyhemoglobin (HHB). However, the authors of the article emphasized that the relationship between these signals should be examined. 

The main objective of this paper was to investigate the relationship between EMG and fNIRS signals from the lower extremities during dynamic movements. The study was supported by experimental results in which an assessment of exercise intensity was performed using two selected protocols (Astrand–Rhyming Step Test [26] and Astrand Treadmill Test [27]). When the results of this study carried out by the authors are compared with the state-of-the-art research, it can be highlighted that the considered research problem is novel. Due to differences in the lifestyles of the participants (sedentary, standing, mixed, and active), the authors of this study decided to include this information in the general characteristics of the participants. During this study, the hypothesis was presented that the type of lifestyle of the participant may influence the correlation coefficient value between the EMG and NIRS signals. The practical application of the proposed methodology provides a wider range of information on the muscle characteristics of the tested individuals, to what extent their physical activity may affect stabilization, and thus the similarity of signal shape in selecting an appropriate test. In the case of this study, the step test (5 min) and the treadmill test (up to 12 min, up to maximum fatigue) were used. A large amount of data recorded using EMG and fNIRS sensors allowed the calculation of average values with standard deviations for each individual, followed by a comparison of the obtained values within the same group of volunteers. The signals analyzed do not consider the fatigue of individual muscles or their activity, but only the relationship between the signals registered in each movement with large fluctuations caused by the displacement of the legs over time. However, the measurement protocols (step test and treadmill) help evaluate signal changes and potentially determine their differences. The results of the tests are presented in this article with a limited number of volunteers. The following approach to this study will be carried out with an appropriate group of volunteers to allow statistical analysis of the results obtained. 

## 2. Materials and Methods

### 2.1. Participants Characteristics

Five women (age 20–35 years, body mass index (BMI) 19–21, and height greater than 160 cm due to step height) volunteered as participants for the present study. Each participant was characterized by a different lifestyle. Details of the characteristics of the participants are presented in Table 1.

All participants were healthy and had no history of serious illness that would affect this study. All participants were fully informed of the procedures and possible risks of the experiment and provided written consent to participate. This study received approval from the Ethics Committee of the Warsaw University of Life Sciences number 19/2022. 

### 2.2. Testing Procedures

Each volunteer who participated in the tests provided the following parameters: weight, height, and age. Before the test procedure, the resting pulse was measured using a pulse oximeter, and this measurement was used to verify the HR measurement from the sensor. Participants performed a 5 min warm-up that included squats, stretching exercises, and crunches, followed by a 10 min recovery. Subsequently, the participants performed the Astrand–Rhyming step test [26]. They had to climb the step at a rhythm of 22.5 times per 60 s for 5 min starting with the right leg (sound signals were used to maintain the rhythm; in case of loss of rhythm, assistance was provided to restore the correct rhythm). The height of the step was 33 cm (the height of the step required for women [26]). HR was measured 15 to 30 s after completing the test and converted to 1 min (that is, the counted number of heart contractions was multiplied by 4). Furthermore, based on the sex and weight of the participants, a nomograph was used to obtain the maximum oxygen consumption (VO2max). The measured pulse and weight of the participants were marked and connected with a line segment. VO2max was obtained from the intersection of this segment with the VO2max scale and multiplied by the appropriate coefficient for the age of the participant. The researchers observed the movements and conditions of the participants during the measurements and recorded their movements on video to verify each activity (after the test/not in real time), and the elimination of possible artifacts depended on the movements.

The second test that was used to determine the maximum oxygen consumption was the Astrand Treadmill Test [27]. It was carried out a few days after the first tests to allow the participants to rest. Resting pulse measurements, a 5 min warm-up, and a 10 min recovery period were implemented as described above. Each participant then ran on a treadmill at a constant speed until exhaustion. The incline was 0% for the first three minutes and then increased by 2.5% after every two minutes of running. The maximum oxygen consumption was determined from the following Formula (1):

(1) $${\mathrm{VO}}_{2\max}\;[\mathrm{mL}/\mathrm{kg}/\min]=(t\times 1.444)+14.99$$

where *t* is the total test time in minutes.

**Figure 1:**
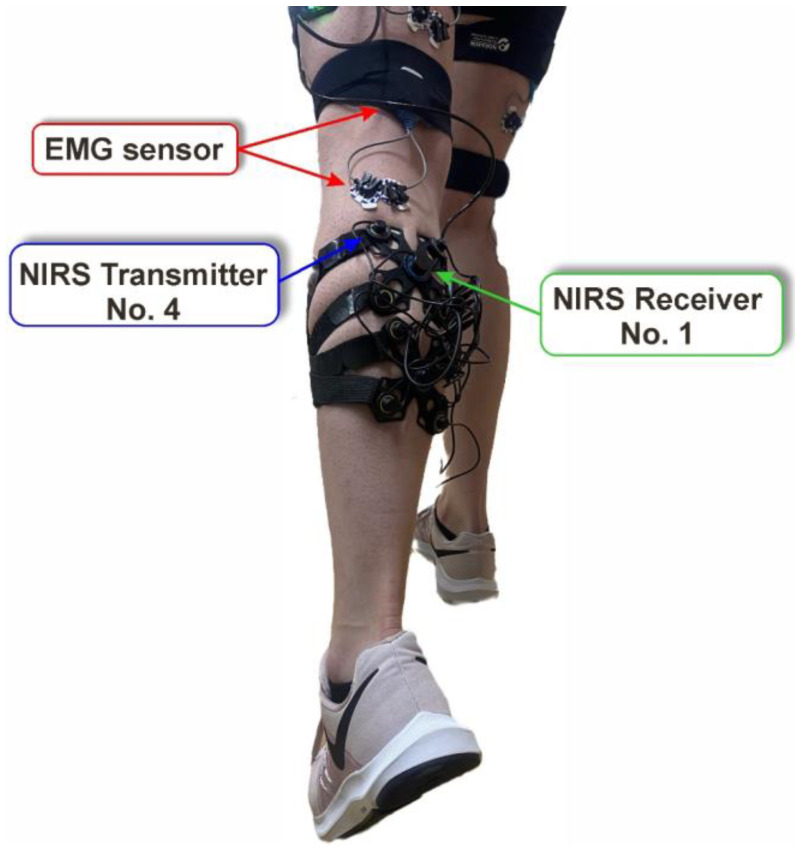
The main view of the proposed testing setup, the EMG sensor, transmitter, and components of the fNIRS system.

To obtain the result in [L/min], VO_2max_ was multiplied by the weight of the patient and divided by 1000. These calculations were performed by the authors due to the test procedures, but they were not important for the main purpose of this study. During the measurements, the authors analyzed the movements and conditions of the participants using recorded movie frames. During the tests, parameters such as oxygen consumption and muscle activation were measured using the EMG sensor and the fNIRS sensor (Figure 1). Pictures of the positions of the devices mounted on the patient’s body were taken to ensure accurate placement in subsequent tests.

An identical acquisition configuration was used in the Astrand–Rhyming Step Test and the Astrand Treadmill Test. In this study, a 32-channel Ultium EMG system (Noraxon, DTS, Desktop Direct Transmission System, Scottsdale, AZ, USA) with a 24 bit sampling rate of 4000 Hz was used to perform measurements. The placement of electrodes on the bodies of the participants was determined according to the needs of the study. fNIRS measurements were acquired with an OctaMon M system (Artinis, LLC, New York, NY, USA). Two receivers and eight transmitter probes were plugged into the holders. The inter-optode distance varied and was less than 3 mm. A dark black cover was applied to the muscle to block external light luminance. The absorption of near-infrared light of two different wavelengths (760.0 and 850.0 nm) was measured. Dedicated software (OxySoft, Elst, The Netherlands) was used for the analysis of the system data, and a Bluetooth device was used to transfer the fNIRS signal directly to the PC computer.

During both tests, the activities of the femoral biceps, triceps of the calf muscle, and quadriceps were subjected to an EMG sensor. The electrodes, which had a center-to-center distance of 20.0 mm, were placed longitudinally in the muscle attachments, which were identified by palpation (on both legs). The sampling rate was set at 2000 Hz, and the 12 channels were connected. EMG activities were measured to verify the maximum activation and the relationships between them during a specific movement. A camera was connected to the EMG Noraxon system, which was essential to perform a precise motion analysis. A pre-analysis was performed to select the dominant muscle in the step and treadmill tests. Based on the recorded video and EMG amplitude, the triceps of the calf muscle were chosen for EMG–fNIRS correlation analysis. Due to the large amount of data and the shorter distance between the sensors (EMG and fNIRS), transmitter No. 4 was chosen.

The Ultium EMG system was used to measure HR, as this parameter was analyzed in both tests (Astrand–Rhyming Step Test and Astrand Treadmill Test). Two electrodes (single electrode pair) placed at the breast generated five different, simultaneous real-time data streams: electrocardiography (ECG), HR, R-R intervals, thoracic impedance (respiration), and respiration rate. Data were registered in the MyoResearch XP Master Edition for DTS Noraxon and synchronized with the EMG activity data.

The OctaMon M is a portable NIRS device optimized for muscle oxygenation measurements. Changes in oxyhemoglobin, deoxyhemoglobin, and total hemoglobin values can be regionally monitored with a 2 × 4 channel configuration. These parameters were recorded during both tests using the fNIRS OctaMon M system, which was placed on the triceps calf muscle. A total of eight channels covering the calf area were used. Each channel width is customizable and can provide a variety of possible settings according to the requirements of sports science research. The adaptable channel widths were 25–40 mm. A sampling rate of 10 Hz was used during the measurements.

### 2.3. Data Analysis

Processing, analysis, and ultimately interpretation of data of dynamic movements generate a number of problems, including distinguishing between individual activities and selecting comparison methods. Data obtained through EMG sensors allows for the determination of the electrical activity of muscles during a movement, whereas fNIRS enables the recording of information on hemoglobin concentration levels. These data show the physiological coherence associated with metabolism. Proper data preparation (filtering and transformation), followed by mutual comparison, provides more information on the changes in signals during human body movements resulting from the activity of individual muscles.

The EMG signal depends on the anatomical properties of the individual and accumulates signals from various motor units. During each of the conducted tests, video recordings of human movements were captured along with the EMG signal. In the case of a step test, the differences in the EMG signal between both legs are due to the different types of movements performed by each leg. To verify the pace of exercise, an analysis of the EMG signal was performed for the right leg (the right leg started the test) using MATLAB. Based on the analysis, a speed of approximately 23 times per minute was determined, which is consistent with the design of the research procedure.

Repeated occurrence of the local maximum of the RMS envelope of the EMG signal was observed during specific test phases (3 phases in the Astrand–Rhyming Step Test and 5 phases in the Astrand Treadmill Test). During the Astrand–Rhyming Step Test, the local maximum of the selected left leg muscles was reached in the kick phase of the left leg time, whereas for the right leg muscles, regularity was observed in the step phase of the right leg time. In the Astrand Treadmill Test, similar correlations were recorded. For each leg, the maximum EMG occurred during the loading phase with full body weight. Data were imported into MATLAB R2022B for further analysis, which confirmed that the absolute activity was highest in the calf muscle. To remove noise, the Butterworth filter was adopted with cutoff frequencies of 20 Hz and 500 Hz, which was used to delimit the physiological frequency band of the EMG signal and to remove high- and low-frequency interference [28].

The fNIRS signal was performed using identical procedures in both tests. The data collected were exported to a text file. Subsequently, the signal was analyzed using MATLAB software. The discrepancies between the signal plots were indicated by one of the NIRS sensors placed on the left calf during the Astrand–Rhyming Step Test.

The key parameter in the Astrand–Rhyming Step Test and the Astrand Treadmill Test is HR. This parameter was continuously measured using the same procedure during both tests. The data were exported to a text file, and then the raw signal was further analyzed in MATLAB. For each test, the mean HR in a specific time range was calculated in MATLAB based on the requirements of the protocol.

## 3. Results

The purpose of this study was to investigate the correlation between the shape of the EMG signal and the shape of the fNIRS signal during exercise and the relationship between the maximum oxygen consumption measured indirectly with HR and the change in local oxygenation in the calf muscle. In the experiment, the synchronized EMG and fNIRS signals were superposed to identify the correlations between them in a single step; a statistical interpretation of the correlation was also provided.

During both tests, the activity and oxygenation of the triceps calf muscle were measured using independent programs. The program switch was manually synchronized. After the scale was adjusted, the changes were observed and summarized collectively. A visible correlation was observed between the shape of the EMG signal and the shape of the fNIRS signal for transmitter No. 4. For the other transmitters, a correlation was observed between the shapes of the fNIRS and EMG signals, but with a slight time change of approximately 0.2 to 0.4 s. The distance between the NIRS transmitter number four and the EMG sensor was approximately 4 cm. The HHb of the fNIRS transmitter was taken in case the EMG amplitude increased. It was caused by a simultaneous increase in the deoxy concentration [25].

Furthermore, to demonstrate the correlation between EMG and NIRS, the Pearson linear correlation coefficient and the Spearman coefficient were calculated. Due to the nature of the data distribution, two approaches were used to determine the correlation for comparison purposes.

The Pearson linear correlation coefficient described by Formula (2) was calculated [29]:

(2) $$r\left(X,Y\right)=\frac{\sum_{i=1}^{N}\left(X_{i}-\overset{-}{X})(Y_{i}-\overset{-}{Y}\right)}{\sqrt{\sum_{i=1}^{N}{\left(X_{i}-\overset{-}{X}\right)}^{2}}\sqrt{\sum_{i=1}^{N}{\left(Y_{i}-\overset{-}{Y}\right)}^{2}}}$$

where *X* is a random variable (EMG), *Y* is a random variable (fNIRS), and *N* is the number of samples.

The Spearman rank order coefficient was calculated based on Pearson’s correlation with the ranged data [30]. The Spearman correlation is used for variables with a non-normal distribution, in contrast to the Pearson correlation, whose normal distribution of the data is recommended [31]. These coefficients are intended to determine the similarity values of the shapes of both signals at each moment of movement. The greater the coefficient, the greater the similarity between the two signals at each moment of movement.

**Figure 2:**
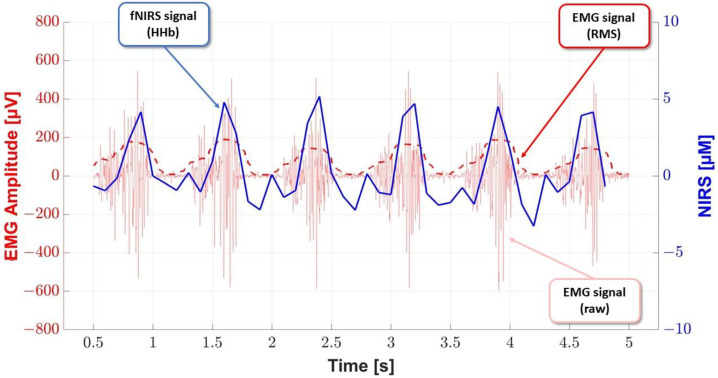
Correlation between the RMS envelope of the EMG signal and deoxyhemoglobin in the selected range.

In the example studied, the random variable *X* is in the RMS envelope of the EMG signal, and the random variable *Y* is deoxyhemoglobin (Figure 2). For the distributions of these random variables, Pearson’s linear correlation coefficient was equal to 0.86, but Spearman’s correlation coefficient was equal to 0.84, indicating a strong positive correlation.

The selected signal fragment showed a high correlation coefficient. However, to verify the correlation in the context of both tests, an analysis of the correlation coefficient values was carried out for all test participants, comparing muscle activity according to the protocol (stepping in and out of the step and bending the leg in the knee while running).

For each participant, the correlation coefficients were determined for each stage of their activity. A stage is a segment of activity that starts every minute and lasts no more than 60 s. For example, if a participant’s activity lasted 470 s, eight stages could be identified (the last lasting 50 s), resulting in a total of eight correlation values. Table 2 and Table 3 present some parameters of the data distribution for the step test and the treadmill test, respectively.

**Figure 3:**
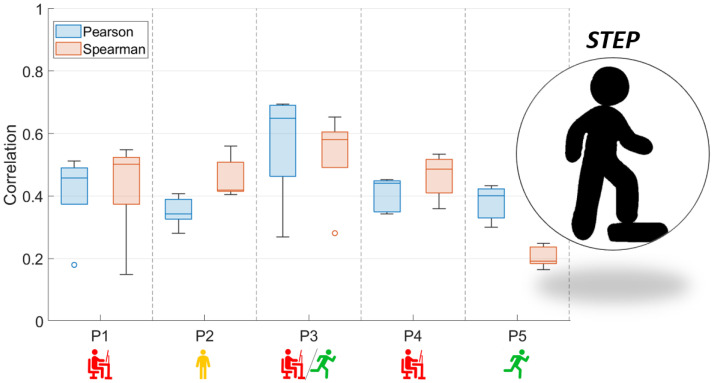
Box and whisker plots of Pearson and Spearman correlations of signals: RMS envelope of the EMG and HHB for STEP (red—sedentary; yellow—standing; red/green—mixed; green—active).

**Figure 4:**
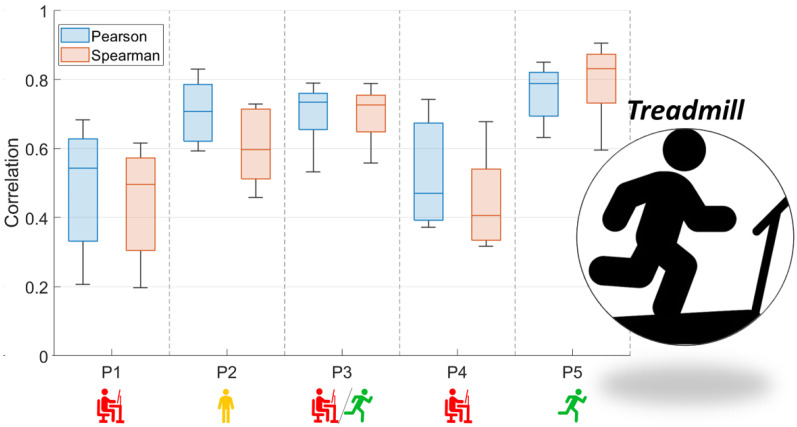
Box and whisker plots of Pearson and Spearman correlations of signals: RMS envelope of the EMG and HHB for TREADMILL (red—sedentary; yellow—standing; red/green—mixed; green—active).

Figure 3 and Figure 4 present the comparison between the correlation values obtained with the use of two functions (Pearson and Spearman), taking into account both analyzed activity tests.

In the case of Figure 3, which is related to the STEP test, the correlation coefficients do not allow the conclusion that there is a relationship between the correlation values of the EMG and NIRS signals and the characteristics of the subjects. This may be due to the adopted form of exercise, which is conditioned by the repetitiveness of moving up and down from the step. In the case of the results presented in Figure 4, the coefficient values obtained were related to their characteristics. As a result, the level of correlation between both signals was found to increase when the participants were used to running.

Four ANOVA tests were performed for the four cases to estimate whether the differences between the correlations of all participants were statistically significant. As all four ANOVA tests showed statistically significant differences, an additional Tukey test was performed to identify pairs of groups that were statistically significantly different.

**Figure 5:**
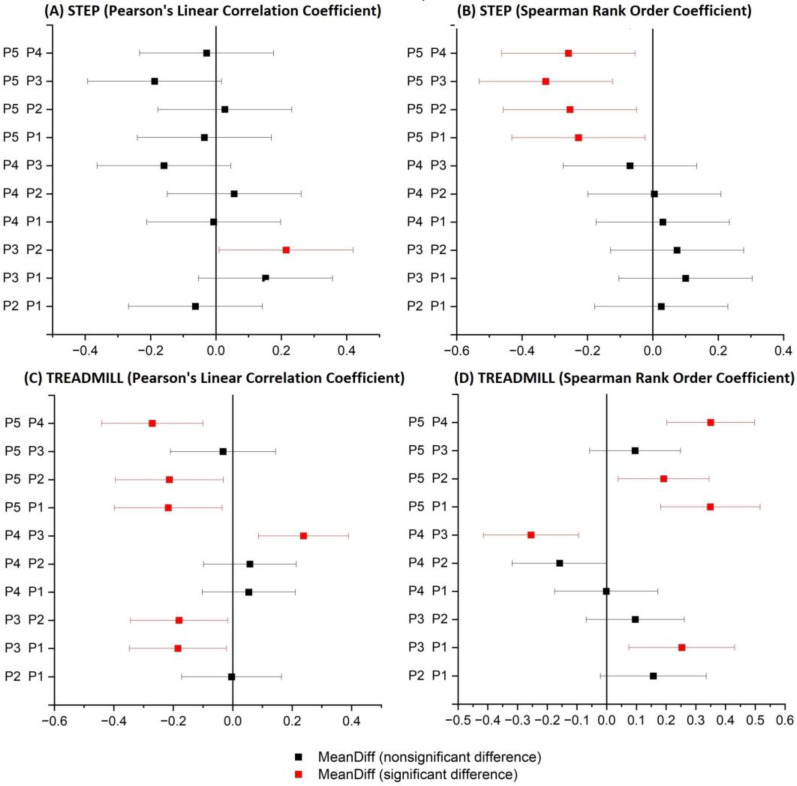
Results of the Tukey test for Pearson and Spearman correlations of signals: RMS envelope of the EMG and HHB for the protocol: Step and Treadmill.

Figure 5 shows the results of the four Tukey tests for the four cases, and red indicates pairs that are statistically significantly different. In the case of the running protocol, these pairs represent participants with different levels of training. In the step protocol, no specific relationship can be demonstrated.

## 4. Discussion

The present study focused on NIRS and EMG measurements of lower limb muscles during two activities: the Astrand–Rhyming Step Test and the Astrand Treadmill Test. Muscle activity and muscle oxygenation were recorded to determine the relationships between EMG and fNIRS signals. In contrast to the published research results described in the introduction of this article, the focus was on comparing the two signals with each other during dynamic movements. Most reference positions for monitoring muscle activity are defined based on the results of medical studies and are related to isometric contraction [20,32]. Many articles refer to the upper extremity, which has a different range of motion and activity than the lower extremity. Generally, studies describing experiments in which simultaneous EMG and NIRS [20,32,33] are used do not focus on correlations during dynamic movements (such as step or treadmill) between EMG and NIRS signals. To address this issue, a procedure was introduced to define the relationship between the signals recorded during dynamic conditions. This paper provides a detailed discussion of the main stages necessary to establish the correlation between these signals.

In this study, the main physical characteristics of the participants, such as sex, age, weight, height, and degree of training, were taken into account. Furthermore, data collected from participants about their lifestyles allowed the hypothesis that the similarity value increases with the degree of training. The step is a type of physical activity that is not a movement performed naturally by study participants and can be performed in almost any age category. The results of the step test did not show any relationships with participant characteristics; however, the coefficient achieved positive values in each case. The situation was different in the case of the treadmill. Lifestyle and the value of the correlation coefficient were associated with each other. For people with the least physical activity (two individuals), the coefficient reached the lowest values. The professional runner had the smallest discrepancy in the similarity of the shapes of both signals in each second of movement. The degree of training suggests that it is a valuable parameter for identifying the correlations between the EMG and fNIRS signals. The Noraxon system was used to measure EMG and HR, whereas the OctamonM system was used to measure NIRS signals. Due to the use of two independent measurement systems, the signal synchronization process was based on knowing the timestamps at which the measurements were initiated. The difference between the timestamps allowed the required correction to be introduced during the preprocessing stage, which is a limitation of this study. However, recording measurements (and thus being able to assess each stage of exercise execution), preparing the test procedure (measurement time, counting the number of movements performed, and measuring heart rate), and preparing participants for the study (warm-up, rest, setting the pace, familiarizing them with the procedure, conducting sports medicine examinations prior to the study, and collecting data on lifestyle) can help reduce the risk of measurement errors.

Another limitation is related to dynamic measurements, which are affected by errors related to continuous motion. A consistent assembly of the equipment is critical. Both tests were carried out primarily in motion, increasing the probability of destabilization of the position of the equipment. Changes in the sensor position during the study (even a few millimeters) can lead to incorrect results. Proper skin preparation and proper sensor placement are also important. Anatomical differences between participants require additional steps to confirm the correct location of the muscle attachments for sensor placement. An important complication is body fat, which negatively affects the signal quality [34]. Muscle oxygenation was recorded using eight NIRS transmitters in this study. However, the signal from transmitter number four (Figure 1) was analyzed only because it was located at the shortest distance from the EMG sensor. Increasing the distance between the NIRS transmitter and the EMG sensor resulted in time shifts and desynchronization of the signals. The psychological aspect is also very important. The concentration and participation of each participant affects the quality of the results and must be considered. Furthermore, it should be noted that both fNIRS and EMG signals are difficult to precisely localize. In the case of fNIRS, this is due to the relatively large area of measurement (up to 3 cm), the depth of light penetration in biological tissues, and variations in skin color that can introduce noise. Furthermore, because of its high sensitivity, EMG is very susceptible to external factors such as large electromagnetic interference or electrostatic discharge.

To achieve the purpose of the investigation, the behavior of the signal was examined in the case of five women, who were similar in terms of age, BMI, absence of injuries, and diseases, in both tests. Limitations related to measurement were also taken into account, and repeatability was tendentiously verified. For such a selected group, the assumptions confirmed that in both the proposed tests (step test and treadmill test), there is a positive correlation. However, higher values were achieved in the treadmill test. Furthermore, it was noticed that in the treadmill test, the overall characteristics of the subjects (lifestyle) influence the correlation value; the more athletic the lifestyle, the higher the correlation coefficient. This is preliminary research designed to study the effect of correlation on dynamic movements. The small number of participants (five women) is one of the main limitations of the study, which means that the results must be interpreted with caution. However, it can be improved by performing a statistical analysis (more than five participants) of EMG advanced signal processing [35] or fNIRS signal processing [36], which further work would be related to and focused on as a fatigue sample. Future research based on the statistical selection of study participants may yield interesting results, and it is also recommended to compare many methods to process the signals before the correlation is calculated. Despite its preliminary nature, this study was carried out in some terms of fNIRS and EMG practices [37,38] and provided abundant information on the relationship during the different training zones, which can be seen through the value of the correlation coefficient in the different stages of fatigue. The relationships between the signals analyzed showed some correlations through the repeatability of the results for dynamic movements (treadmill and step tests). Test protocols can be used to facilitate the subsequent analysis of results (such as counting muscle activity and identifying movement) and might obtain data on the characteristics of participants and on the correct placement of equipment.

## 5. Conclusions

This study aimed to investigate the relationship between EMG and fNIRS signals during dynamic movements, specifically during the Astrand–Rhyming Step Test and the Astrand Treadmill Test. The authors of this publication focused on investigating the correlation between EMG and NIRS signals of the lower limb during dynamic movements, which has not been the focus of previous articles that describe experiments using simultaneous EMG and NIRS. 

Based on the proposed methodology, the conducted tests, and the results obtained, the following conclusions were formulated:

- The proposed methodology allows for the correlation of both signals during dynamic movements. This approach has been published in a rather limited number of papers.
- The achievement of positive correlations between electrical muscle activity and local muscle oxygenation have been achieved.
- The lifestyles of participants may have an impact on the level of correlation between EMG and NIRS signals.
- During the analysis of data from the treadmill test, it was observed that the more active the participant’s lifestyle, the stronger the correlation between the EMG and NIRS signals. However, this relationship was not observed in the step test case.

Despite some limitations related to the measurement procedure, such as the risk of data abnormalities resulting from device destabilization and the relatively small number of participants, the results of this study contribute to a better understanding of the relationship between EMG and fNIRS signals during dynamic movements. More studies with larger sample sizes and more diverse populations are needed to validate the findings of this study. 

## Figures and Tables

**Table 1:** Participant characteristics.

Participants	Lifestyles	
Participant #1	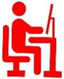	Sedentary lifestyle
Participant #2	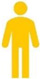	Lifestyle of standing, small activity
Participant #3	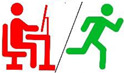	Mixed lifestyle, stressful work, sport after work
Participant #4	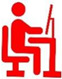	Sedentary lifestyle
Participant #5	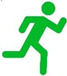	Active lifestyle

**Table 2:** Comparison of the correlation distribution parameters for each participant during the step test.

Participant	N. of Stages	Correlation	MIN	MAX	Q1	Q2	Q3
P1	5	Pearson	0.180	0.512	0.309	0.458	0.497
		Spearman	0.149	0.548	0.299	0.502	0.532
P2	5	Pearson	0.281	0.407	0.311	0.343	0.396
		Spearman	0.406	0.560	0.412	0.419	0.526
P3	5	Pearson	0.269	0.695	0.398	0.650	0.692
		Spearman	0.281	0.654	0.421	0.580	0.622
P4	5	Pearson	0.343	0.453	0.347	0.441	0.450
		Spearman	0.359	0.535	0.393	0.486	0.523
P5	5	Pearson	0.300	0.433	0.320	0.401	0.427
		Spearman	0.165	0.248	0.177	0.192	0.241

**Table 3:** Comparison of the correlation distribution parameters for each participant during the treadmill test.

Participant	N. of Stages	Correlation	MIN	MAX	Q1	Q2	Q3
P1	6	Pearson	0.207	0.682	0.301	0.544	0.642
		Spearman	0.198	0.615	0.278	0.496	0.584
P2	8	Pearson	0.593	0.830	0.619	0.707	0.792
		Spearman	0.459	0.729	0.503	0.597	0.718
P3	8	Pearson	0.532	0.790	0.654	0.734	0.761
		Spearman	0.557	0.787	0.643	0.726	0.760
P4	9	Pearson	0.372	0.742	0.387	0.470	0.691
		Spearman	0.317	0.678	0.332	0.406	0.568
P5	11	Pearson	0.631	0.850	0.691	0.788	0.825
		Spearman	0.596	0.905	0.722	0.832	0.875

## Data Availability

Not applicable.
